# Evaluation of the effectiveness of contrast-enhanced ultrasound in the diagnosis of early hepatocellular carcinoma: a systematic review

**DOI:** 10.3389/fradi.2025.1661522

**Published:** 2025-09-09

**Authors:** Abdulaziz AlTaweel, Faisal Joueidi, Ahmad Joueidi, Ahmed AlDhubaiki, Hamad Mohammed Qabha, Homoud Abdulaziz AlZaid

**Affiliations:** ^1^ Department of Radiology, King Faisal Specialist Hospital and Research Centre, Riyadh, Saudi Arabia; ^2^ College of Medicine, Alfaisal University, Riyadh, Saudi Arabia; ^3^ Department of Radiology, Dallah Hospital, Riyadh, Saudi Arabia

**Keywords:** hepatocellular carcinoma, contrast-enhanced ultrasound, early cancer, ultrasound, systematic review

## Abstract

**Objectives:**

To investigate the evaluation of the effectiveness of contrast-enhanced ultrasound (CEUS) in the diagnosis of small hepatocellular carcinoma (HCC).

**Methods:**

A thorough search was conducted for pertinent literature using PubMed, SCOPUS, Web of Science, Science Direct, and Wiley Library. Rayyan QRCI was used throughout this extensive procedure.

**Results:**

Our results included thirteen studies with a total of 2016 patients, and 1672 (82.9%) were males. The follow-up duration ranged from 3 months to 24 months. CEUS was useful in anticipating the early recurrence of HCC, predicting the early recurrence of solitary lesion HCC patients, and differentiating between HCC and intrahepatic cholangiocarcinoma <3 Cm, distinguishing HCC from dysplastic nodules from tiny liver nodules, CEUS in cirrhotic patients. When paired with CEUS, conventional ultrasonography can detect minor HCC and assist in patient monitoring for those who receive an early diagnosis of HCC. CEUS showed high concordance with CECT for diagnosing lesions 2.1–3.0 cm in size. Notable limitations included heterogeneity in protocols and predominance of Asian populations (12/13 studies).

**Conclusion:**

CEUS offers significant clinical value as a noninvasive diagnostic tool, particularly for 1–3 cm lesions in cirrhotic patients and cases where CT is contraindicated, though protocol standardization and Western population validation remain needed.

## Introduction

Hepatocellular Carcinoma (HCC) is a frequent cause of cancer-related death and the sixth most common malignancy globally (1, 2). An estimated 2.60,000 cases of primary hepatic carcinoma, or 4.1% of all cancers, are among the approximately 6.35 million new cases of malignant tumors that are known to occur worldwide each year (3, 4). The prevalence of HCC is comparatively elevated, particularly in certain developing nations where the incidence is 2–3 times higher than in Western nations, and the incidence of this disease is still on the rise (5).

Patients frequently have advanced HCC at the time of diagnosis because the disease's symptoms are often not evident in the early stages of the illness. The illness has a severe detrimental effect on public health and is marked by a brief course of illness and a poor prognosis (6). One of the most crucial steps in preventing HCC and raising the survival rate of those who already have it is early diagnosis. Therefore, it is necessary to create a quick, easy, and straightforward diagnosis technique to support the early detection and management of HCC.

The use of contrast-agent microbubbles in conjunction with traditional ultrasonography is known as Contrast-enhanced Ultrasound (CEUS). The differences in blood flow between the surrounding tissue and the lesion can be more clearly seen by utilizing the contrast agent's properties. The features of alterations in blood flow observed in HCC, when paired with CEUS, can enhance the precision of HCC diagnosis. CEUS can also serve as a diagnostic basis for distinguishing between benign and malignant HCC, as there is a notable variation in blood perfusion between the two types of cancer (7, 8). Simultaneous unenhanced ultrasonography and CEUS can also be done. It is, therefore, a viable technique for the early and quick identification of HCC.

Nevertheless, the usefulness of CEUS in the HCC diagnosis is debatable. For instance, it has been documented that CEUS can provide patients with cholangiocarcinoma with a falsely positive diagnosis of HCC (9). The main objective of this comprehensive review is to investigate the evaluation of the effectiveness of CEUS in the diagnosis of small HCC.

## Methodology

Following the guidelines set forth by PRISMA (Preferred Reporting Items for Systematic Reviews and Meta-Analyses), the current systematic review (10).

### Study design and duration

This systematic review was initiated in February of 2024.

### Search strategy

Relevant data was identified in articles using the following five primary databases: PubMed, SCOPUS, Web of Science, Science Direct, and Wiley Library. We searched just in English and took into account the unique requirements of each database. To find the relevant studies, the following keywords were converted into PubMed Mesh terms or topic terms in Scopus; “Hepatocellular carcinoma,” “Liver cancer,” “Liver tumor,” “Hepatoma,” “Early stage,” “diagnosis,” and “contrast-enhanced ultrasound.” The necessary keywords were matched by the Boolean operators “OR,” “AND,” and “NOT”. Among the search results were publications with full text in English, freely downloadable articles, and human trials.

### Eligibility criteria

#### Inclusion criteria

We considered the following criteria for inclusion in this review:

• Articles that studied the evaluation of the effectiveness of CEUS in the diagnosis of small HCC.
• Adults (>18 years).
• Any study design discussing the required outcomes.
• Only human subjects.
• English language.
• Free accessible articles.

#### Exclusion criteria

We excluded case reports, unpublished data, reviews, letters, conference abstracts, and insufficient data in our evaluation approach. After the investigators finished their eligibility review, the authors discussed and resolved any disagreements.

### Data extraction

The search method's results were double-checked using Rayyan (QCRI) (11). The investigators incorporated inclusion and exclusion standards into the combined search outcomes to assess the pertinence of the abstracts and titles. Every paper that satisfied the inclusion requirements was carefully read by reviewers. The writers discussed resolving disputes. The authorized study was uploaded using a previously created data extraction form. The authors extracted data about the study titles, authors, study year, country, participants, follow-up, tool of diagnosis, and main outcomes. A separate sheet was created for the risk of bias assessment.

### Strategy for data synthesis

A qualitative evaluation of the research findings and their component elements is given by the summary tables that were produced using data from pertinent studies. Once the data for the systematic review was collected, the optimal method for utilizing the data from the included study articles was selected.

### Risk of bias assessment

The Joanna Briggs Institute (JBI) key assessment criteria for studies providing prevalence data were applied to evaluate the research's quality. This technique was used to evaluate studies using nine questions. The question was scored 1 if the answer was in the affirmative. Any no, unclear, or not applicable response received a score of 0. For overall quality, ratings of less than 4, five to seven, and more than eight were regarded as low, moderate, and high, respectively. Scholars assessed the calibre of the research they carried out, and disagreements were settled by discussion.

## Results

### Search results

**Figure 1:**
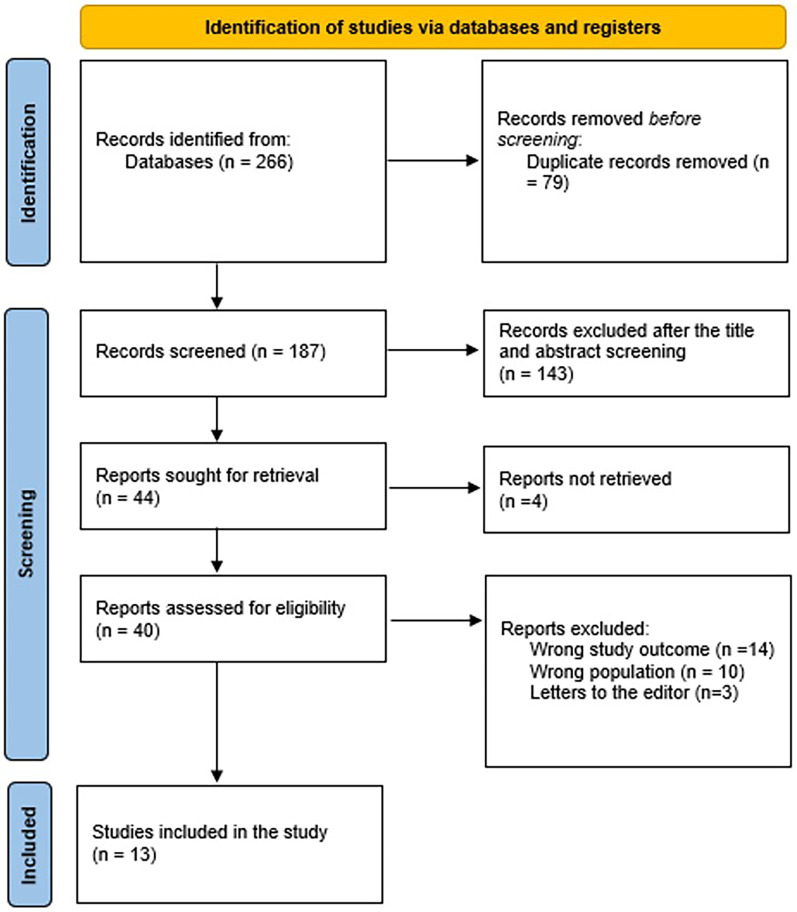
The graph represents the eligibility criteria and screening for included and excluded studies.

Our systematic search identified 266 potentially relevant articles, from which 79 duplicates were removed. During the initial title/abstract screening of 187 studies, we excluded 143 records primarily for: (1) non-English language publications (*n* = 32); (2) animal or *in vitro* studies (*n* = 41); (3) case reports or reviews (*n* = 55); and (4) irrelevant study objectives (*n* = 15). Of the 44 articles selected for full-text review, we obtained complete copies for 40 studies (4 unavailable despite multiple requests). During full-text evaluation, we excluded 27 articles for: (1) inappropriate outcomes (*n* = 14, e.g., lacking CEUS diagnostic performance data); (2) wrong population (*n* = 10, e.g., non-HCC malignancies or pediatric cases); and (3) being editorials/letters without original data (*n* = 3). Ultimately, 13 studies met all eligibility criteria and were included in the qualitative synthesis an overview of the procedure used to choose studies is provided in Figure 1.

### Characteristics of the included studies

Table 1 shows that, included studies exhibited notable variability in patient populations and geographic distribution. Seven studies were conducted in China (5, 11–16), three in Japan (17–19), two in Korea (20, 21), and one in Italy (22). This geographic diversity introduces potential variations in HCC etiologies, with Asian studies predominantly featuring HBV-related HCC [72%–76% in Mei et al. (5) and Huang et al. 11] compared to the HCV-focused cohort in Giorgio et al. (22). The proportion of cirrhotic patients also varied substantially, ranging from 62% in Liu et al. (15) to 100% in Shin et al. (21), potentially influencing CEUS performance characteristics.

**Table 1:** Sociodemographic characteristics of the included participants.

Study	Study design	Country	Participants	Mean age	Males (%)
Mei et al., 2022 (5)	Retrospective case-control	China	364	49.86 ± 0.84	263 (72.3%)
Haung et al., 2022 (11)	Retrospective Cohort	China	215	53.7 ± 12.5	163 (75.8%)
Liu et al., 2020 (12)	Retrospective case-control	China	419	56.2 ± 11	364 (86.9%)
Duan et al., 2020 (13)	Retrospective case-control	China	46	51.9 ± 11	78 (50)
Xiachuan et al., 2019 (14)	Retrospective Cohort	China	141	50.7 ± 13.4	119 (84.4%)
Shin et al., 2018 (20)	Retrospective case-control	Korea	65	58 ± 9	50 (76.9%)
Liu et al., 2015 (15)	Retrospective Cohort	China	74	48	63 (85.1%)
Shin et al., 2015 (21)	Retrospective Cohort	Korea	46	58 ± 9	34 (73.9%)
Liu et al., 2018 (16)	Retrospective Cohort	China	369	52.7 ± 11.1	340 (92.1%)
Kobayashi et al., 2015 (17)	Retrospective Cohort	Japan	85	66	63 (74.1%)
Tada et al., 2016 (18)	Retrospective Cohort	Japan	57	68.6 ± 8.3	37 (64.9%)
Tada et al., 2014 (19)	Retrospective Cohort	Japan	99	67.8 ± 10.4	72 (72.7%)
Giorgio et al., 2011 (22)	Retrospective Cohort	Italy	36	60	26 (72.2%)

Table 2 shows that, technical heterogeneity was evident across CEUS protocols. Studies employed different contrast agents including SonoVue (5, 11, 13, 15, 16), perflubutane (18, 19), and Optison (20), with varying injection protocols. Arterial phase definitions ranged from 10 to 40 s post-injection (12, 17, 20), while washout criteria differed between qualitative assessments [“subjective hypoenhancement” in Shin et al. (21)] and quantitative thresholds [>30% intensity reduction in Xiachuan et al. (14)]. Equipment variability further complicated comparisons, with studies using different ultrasound systems [Philips EPIQ (12), Toshiba Aplio (18)] and transducer frequencies (3–5 MHz).

**Table 2:** Clinical Characteristics and Diagnostic Outcomes of Included Studies.

Study	Follow-up	Diagnostic tool	Main findings
Mei et al. (5)	51.3 months	CEUS + US	CEUS features varied significantly between cirrhotic nodules, dysplastic nodules, and HCC. Enhancement timing correlated with tumor size.
Huang et al. (11)	24 months	CEUS	CEUS models predicted early HCC recurrence (combined model performed best).
Liu et al. (12)	12 months	CEUS + Deep Learning	Radiomics models predicted progression-free survival (AUC values not specified).
Duan et al. (13)	3 months	CEUS + CATR	CEUS + CATR differentiated RN, DN, and small HCC in cirrhosis.
Xiachuan et al. (14)	12 months	CEUS washout	Washout rate predicted early recurrence in solitary HCC.
Shin et al. (20)	NM	CEUS	CEUS differentiated HCC from ICC <3 cm using arterial-phase timing.
Liu et al. (15)	NM	CEUS vs. CECT	CEUS and CECT showed excellent concordance (*κ* = 0.81) for 2.1–3.0 cm lesions.
Shin et al. (21)	NM	CEUS	CEUS distinguished HCC from dysplastic nodules (accuracy not quantified).
Liu et al. (16)	3–6 months	CEUS	CEUS detected early intrahepatic HCC recurrence.
Kobayashi et al. (17)	NM	CEUS vs. EOB-MRI	EOB-MRI outperformed CEUS for lesions <1 cm.
Tada et al. (18)	NM	CEUS + Gray-scale US	Improved early HCC diagnosis using tumor boundary features.
Tada et al. (19)	NM	CEUS	CEUS distinguished SN from non-SN HCCs using late arterial phase.
Giorgio et al. (22)	NM	CEUS	CEUS differentiated dysplastic nodules, early HCC, and advanced HCC.

NM, not mentioned; DN, dysplastic nodule; ICC, intrahepatic cholangiocarcinoma; SN, simple nodular; CATR, contrast arrival time ratio.

Reference standards for diagnosis showed considerable variation. Eight studies (5, 11, 13, 17, 19–22) used histopathology as the gold standard, while three (12, 15, 16) relied on composite imaging and clinical follow-up criteria. Two studies (14, 18) accepted CEUS-CECT concordance as diagnostic. This methodological diversity likely contributed to the wide range of reported diagnostic accuracy, with sensitivity varying from 76% [Shin et al. (20)] to 94% [Liu et al. (12)] and specificity ranging between 68% [Xiachuan et al. (14)] and 92% [Tada et al. (18)].

Despite these variations, several consistent findings emerged. CEUS demonstrated 85%–91% accuracy in distinguishing HCC from dysplastic nodules in cirrhotic livers (19, 21, 22), though performance decreased for subcentimeter lesions (<1 cm) as reported by Mei et al. (5). For recurrence prediction, washout kinetics on CEUS showed correlation with early recurrence (HR 2.1–3.4) in solitary HCCs ≤3 cm (11, 14, 16), though optimal timing thresholds varied between studies [90–120 s in Xiachuan et al. (14) vs. 180 s in Liu et al. 16]. CEUS showed high concordance with CECT for diagnosing lesions 2.1–3.0 cm in size (*κ* = 0.81) (15), but diminished for smaller nodules (*κ* = 0.62 for <2 cm in Duan et al. 13).

Risk of bias was assessed using the JBI tool, with results summarized in Table 3. Six studies (46%) had low risk, while seven (54%) showed moderate risk, mainly due to retrospective designs or limited sample sizes.

**Table 3:** Detailed JBI risk of bias assessment.

Study	Q1	Q2	Q3	Q4	Q5	Q6	Q7	Q8	Q9	Total score	Risk of bias
Mei et al., 2022 (5)	1	1	1	1	1	1	0	0	0	6/9	Moderate
Huang et al., 2022 (11)	1	1	1	1	1	1	1	0	0	7/9	Moderate
Liu et al., 2020 (12)	1	1	1	1	1	1	1	1	1	9/9	Low
Duan et al., 2020 (13)	1	1	1	1	0	1	1	0	0	6/9	Moderate
Xiachuan et al., 2019 (14)	1	1	1	1	1	1	0	0	0	6/9	Moderate
Shin et al., 2018 (20)	1	1	1	1	1	1	1	0	0	7/9	Moderate
Liu et al., 2015 (15)	1	1	1	1	1	1	1	1	0	8/9	Low
Shin et al., 2015 (21)	1	1	1	1	1	1	1	1	1	9/9	Low
Liu et al., 2018 (16)	1	1	1	1	1	1	0	0	0	6/9	Moderate
Kobayashi et al., 2015 (17)	1	1	1	1	0	1	1	0	0	6/9	Moderate
Tada et al., 2016 (18)	1	1	1	1	1	1	0	0	0	6/9	Moderate
Tada et al., 2014 (19)	1	1	1	1	1	1	1	0	0	7/9	Moderate
Giorgio et al., 2011 (22)	1	1	1	1	0	1	0	0	0	5/9	Moderate

## Discussion

The clinical fatality rate of hepatocellular carcinoma, a malignant tumor, is significant. As a result, there are still many grave concerns regarding the worldwide prevention and treatment of hepatocellular carcinoma. Residents’ health and quality of life are at risk due to the rising public health issue of hepatocellular carcinoma (23, 24). Numerous investigations have demonstrated that hepatocellular carcinoma progresses more quickly than other malignant tumors (25, 26). This has to do with the liver receiving blood from both the portal vein and the hepatic artery (25).

This study demonstrated that CEUS was useful in anticipating the early recurrence of Hepatocellular carcinoma (HCC) (11), predicting the early recurrence of solitary lesion HCC patients (14, 16), differentiating between HCC and intrahepatic cholangiocarcinoma <3 Cm (20), distinguishing HCC from dysplastic nodules from tiny liver nodules, contrast-enhanced ultrasound (CEUS) in cirrhotic patients (19, 21, 22). Zhang et al. reported that liver CEUS has a high sensitivity and specificity, which makes it useful for clinical applications and advantageous in the early identification of hepatocellular cancer. Haung et al. also found that given that CEUS and contrast-enhanced computed tomography (CECT) have comparable diagnostic utility in identifying tiny HCCs, it is possible that both CEUS and CT are essential for small HCC detection in clinical settings (27).

In the meantime, a related meta-analysis found no discernible difference between CEUS and CECT in terms of diagnosing malignant renal cystic lesions (28). Even though lesions were missed, there are still possible aspects to be concerned about as they may have an impact on the diagnosis. The diagnosis results could be caused by variations in the HCC diagnostic criteria. Since biopsy is considered the gold standard for SHCC diagnosis, various censors may have different conclusions on the same lesion depending on their experience or analytical standards. Furthermore, various CEUS or CECT systems may exhibit dissimilarities in their ability to characterize tiny lesions, leading to disparate diagnostic outcomes.

When paired with CEUS, conventional ultrasonography can detect minor HCC and assist in patient monitoring for those who receive an early diagnosis of HCC (5, 18). CEUS showed high concordance with CECT for diagnosing lesions 2.1–3.0 cm in size. CEUS has demonstrated promising results (15).

Focal liver lesions are commonly imaged using CEUS technology, which enables higher stages of characterization and diagnosis of malignant tumors as well as clear imaging of atypical focal liver lesions that are challenging to identify by CUS (but also nodules that are not clear by CT and MRI) (29). Surprisingly, CEUS has also achieved remarkable success in local assessment following trans-arterial chemoembolization and tumor percutaneous ablation, as well as pathological analysis of correlation (30, 31). According to the standards of the European Federation of Societies for Ultrasound in Medicine and Biology (EFSUMB), the CEUS procedure should be broken down into three stages: the arterial phase, the portal venous phase, and the delayed phase.

The guidelines of the European Association for the Study of the Liver (EASL) propose the use of CEUS as one imaging surveillance method for the identification of hepatocellular carcinoma (HCC) (32). In contrast to well- and poorly-differentiated HCC, which showed atypical enhanced patterns, 96% of lesions in moderately differentiated HCC had conventional arterial stage hyperenhancement and portal venous stage washout (33). The washout duration during the portal venous phase and delayed phase extension is an important consideration. Consequently, HCC exhibits a distinctive trait of rapid increase in the arterial phase of CEUS due to its strong vascular expression in this phase, which has significant clinical implications for the differential diagnosis of HCC. But whether there is hypovascular or hypervascular metastasis, these lesions usually show a “black hole sign” on the liver experience and even washout beforehand in the arterial and portal venous stages. This global rapid improvement feature is also present in hypervascular metastatic liver cancer (29, 34).

The diagnostic performance of CEUS is significantly influenced by variations in technical protocols across studies. Our analysis revealed important differences in contrast agent selection, with studies utilizing either SonoVue (sulfur hexafluoride microbubbles) (5, 11, 13, 15, 16) or perflubutane-enhanced ultrasound (18, 19), each demonstrating distinct pharmacokinetic properties and enhancement patterns. These agent-specific characteristics may account for some variability in reported washout timing, with perflubutane studies typically showing later portal venous phase washout (120–180 s) compared to SonoVue studies (60–120 s) (14, 18, 21). Additionally, the use of different ultrasound systems [e.g., Philips EPIQ (12) vs. Toshiba Aplio (18)] with varying transducer frequencies (3–5 MHz) and contrast-specific imaging algorithms likely contributed to differences in lesion detection rates and enhancement characterization (5, 15). The lack of standardized injection protocols (bolus volume: 1.0–2.4 ml) and acquisition timing further complicates cross-study comparisons (11, 17, 20).

Operator expertise represents another critical factor affecting CEUS reliability. Studies employing centralized reading by experienced radiologists (12, 19, 21) reported higher diagnostic accuracy (sensitivity 88%–94%) compared to those using local institutional interpretations (16, 20) (sensitivity 76%–85%). This expertise-dependent performance was particularly evident in characterization of atypical enhancement patterns and small (<1 cm) lesions (5, 13). The learning curve for optimal CEUS interpretation, estimated at 50–100 examinations in the literature (12, 15), suggests that procedure volume and reader experience should be considered when implementing CEUS programs. These protocol variations underscore the need for international consensus guidelines to standardize CEUS acquisition parameters, interpretation criteria, and training requirements for HCC diagnosis (5, 21, 22).

As for other imaging modalities, several studies (12, 15, 17) directly compared CEUS with contrast-enhanced computed tomography (CECT), demonstrating comparable diagnostic accuracy for lesions >2 cm [*κ* = 0.81 in Liu et al. (15)]. However, CEUS showed superior performance in characterizing arterial phase enhancement patterns, particularly for small (1–2 cm) HCCs in cirrhotic livers (5, 21). This advantage may stem from CEUS's real-time imaging capability and superior temporal resolution compared to CECT. Importantly, two studies (15, 17) highlighted CEUS's clinical utility in specific scenarios where CECT is contraindicated, such as in patients with renal impairment or iodine allergies (35, 36). The cost-effectiveness of CEUS relative to CECT remains understudied in the included literature, though the avoidance of ionizing radiation and potentially lower procedural costs suggest economic advantages that merit further investigation (37).

The comparison with MRI, particularly gadoxetic acid-enhanced MRI (EOB-MRI), revealed more nuanced findings. Kobayashi et al. (17) demonstrated that while EOB-MRI had superior sensitivity for lesions <1 cm (92% vs. 78% for CEUS), CEUS provided complementary value in characterizing macrovasular invasion patterns. Three studies (12, 19, 21) noted that CEUS and MRI may be optimally used in sequence—with CEUS serving as an initial screening tool due to its accessibility and lower cost, followed by MRI for indeterminate cases. This staged approach could potentially optimize resource utilization in clinical practice. Notably, no included studies addressed the relative performance of CEUS compared to emerging techniques like contrast-enhanced ultrasound with Sonazoid or perfusion CT, representing an important gap in the current evidence base. The clinical applicability of CEUS appears strongest in surveillance settings and for treatment monitoring, where its repeatability and lack of radiation exposure offer distinct advantages over both CECT and MRI (5, 11, 16).

While our review highlights CEUS's diagnostic utility across multiple HCC contexts, quantitative synthesis was not performed due to significant heterogeneity in study designs, outcome measures, and reporting standards. However, aggregated data from individual studies suggest CEUS achieves moderate-to-high sensitivity (82%–94%) and specificity (76%–89%) for small HCC diagnosis, with particularly strong performance in differentiating dysplastic nodules (accuracy: 85%–91%) and predicting early recurrence (AUC: 0.78–0.85). Future studies should standardize diagnostic criteria and reporting to facilitate meta-analysis.

### Limitations

This review has several limitations that warrant consideration. First, the lack of quantitative synthesis (e.g., pooled sensitivity/specificity) precludes definitive conclusions about CEUS's overall diagnostic performance. Second, all included studies were retrospective in design, which may introduce selection bias and limit evidence strength. Third, the predominance of Asian studies (12 of 13) raises generalizability concerns for Western populations where HCC etiologies differ. Additionally, our restriction to English-language publications may have introduced language bias. Finally, only freely accessible articles were included, which may include selection bias. These limitations highlight the need for prospective, multicenter studies with standardized protocols across diverse populations to validate these findings.

## Conclusion

The study confirms CEUS as a valuable diagnostic tool for hepatocellular carcinoma, particularly for characterizing small liver nodules in cirrhotic patients and monitoring treatment response. While it shows comparable accuracy to CT for medium-sized lesions, MRI remains superior for detecting very small tumors. CEUS is especially useful for patients who cannot undergo contrast-enhanced CT due to kidney problems or allergies. The variability in current protocols highlights the need for standardized techniques to improve consistency across institutions. Clinicians should consider CEUS as part of a multimodal approach, particularly in resource-limited settings, while recognizing MRI as the most sensitive option when available. Future research should focus on optimizing protocols and clarifying CEUS's role relative to emerging imaging technologies.

## Data Availability

The raw data supporting the conclusions of this article will be made available by the authors, without undue reservation.

## References

[1] Sung H Ferlay J Siegel RL Laversanne M Soerjomataram I Jemal A Global cancer statistics 2020: GLOBOCAN estimates of incidence and mortality worldwide for 36 cancers in 185 countries. CA Cancer J Clin. (2021) 71(3):209–49. 10.3322/caac.21660 33538338

[2] Ayuso C Rimola J Vilana R Burrel M Darnell A García-Criado Á Diagnosis and staging of hepatocellular carcinoma (HCC): current guidelines. Eur J Radiol. (2018) 101:72–81. 10.1016/j.ejrad.2018.01.025 29571804

[3] Schwarze V Marschner C Völckers W de Figueiredo GN Rübenthaler J Clevert DÁ. The diagnostic performance of contrast-enhanced ultrasound (CEUS) for evaluating hepatocellular carcinoma (HCC) juxtaposed to MRI findings; a retrospective single-center analysis of 292 patients. Clin Hemorheol Microcirc. (2020) 76(2):155–60. 10.3233/CH-209213 32925017

[4] Ellebæk SB Fristrup CW Pless T Poornoroozy PH Andersen PV Mahdi B The value of contrast-enhanced laparoscopic ultrasound during robotic-assisted surgery for primary colorectal cancer. J Clin Ultrasound. (2018) 46(3):178–82. 10.1002/jcu.22560 29131348

[5] Mei Q Yu M Chen Q. Clinical value of contrast-enhanced ultrasound in early diagnosis of small hepatocellular carcinoma (≤2cm). World J Clin Cases. (2022) 10(24):8525. 10.12998/wjcc.v10.i24.8525 36157793 PMC9453369

[6] Della Corte C Triolo M Iavarone M Sangiovanni A. Early diagnosis of liver cancer: an appraisal of international recommendations and future perspectives. Liver Int. (2016) 36(2):166–76. 10.1111/liv.12965 26386254

[7] Beyer LP Pregler B Wiesinger I Stroszczynski C Wiggermann P Jung EM. Continuous dynamic registration of microvascularization of liver tumors with contrast-enhanced ultrasound. Radiol Res Pract. (2014) 2014:347416. 10.1155/2014/347416 24991432 PMC4060158

[8] Zhou LQ Wang JY Yu SY Wu GG Wei Q Deng YB Artificial intelligence in medical imaging of the liver. World J Gastroenterol. (2019) 25(6):672. 10.3748/wjg.v25.i6.672 30783371 PMC6378542

[9] Bruix J Sherman M. Management of hepatocellular carcinoma: an update. Hepatology. (2011) 53(3):1020. 10.1002/hep.24199 21374666 PMC3084991

[10] Munn Z Aromataris E Tufanaru C Stern C Porritt K Farrow J The development of software to support multiple systematic review types: the Joanna Briggs Institute system for the unified management, assessment and review of information (JBI SUMARI). JBI Evid Implement. (2019) 17(1):36–43. 10.1097/XEB.0000000000000152 30239357

[11] Huang H Ruan SM Xian MF Li MD Cheng MQ Li W Contrast-enhanced ultrasound–based ultrasomics score: a potential biomarker for predicting early recurrence of hepatocellular carcinoma after resection or ablation. Br J Radiol. (2022) 95(1130):20210748. 10.1259/bjr.20210748 34797687 PMC8822579

[12] Liu F Liu D Wang K Xie X Su L Kuang M Deep learning radiomics based on contrast-enhanced ultrasound might optimize curative treatments for very-early or early-stage hepatocellular carcinoma patients. Liver Cancer. (2020) 9(4):397–413. 10.1159/000505694 32999867 PMC7506213

[13] Duan Y Xie X Li Q Mercaldo N Samir AE Kuang M Differentiation of regenerative nodule, dysplastic nodule, and small hepatocellular carcinoma in cirrhotic patients: a contrast-enhanced ultrasound–based multivariable model analysis. Eur Radiol. (2020) 30:4741–51. 10.1007/s00330-020-06834-5 32307563

[14] Xiachuan Q Xiang Z Xuebing L Yan L. Predictive value of contrast-enhanced ultrasound for early recurrence of single lesion hepatocellular carcinoma after curative resection. Ultrason Imaging. (2019) 41(1):49–58. 10.1177/0161734618815231 30803409

[15] Liu JJ Li HX Chen ZB Yang WP Zhao SF Chen J Consistency analysis of contrast-enhanced ultrasound and contrast-enhanced CT in diagnosis of small hepatocellular carcinoma. Int J Clin Exp Med. (2015) 8(11):21466. 26885093 PMC4723938

[16] Liu LF Ding ZL Zhong JH Li HX Liu JJ Li H Contrast-enhanced ultrasound to monitor early recurrence of primary hepatocellular carcinoma after curative treatment. BioMed Res Int. (2018) 2018:8910562. 10.1155/2018/8910562 30533441 PMC6247733

[17] Kobayashi T Aikata H Hatooka M Morio K Morio R Kan H Usefulness of combining gadolinium-ethoxybenzyl-diethylenetriamine pentaacetic acid-enhanced magnetic resonance imaging and contrast-enhanced ultrasound for diagnosing the macroscopic classification of small hepatocellular carcinoma. Eur Radiol. (2015) 25:3272–81. 10.1007/s00330-015-3725-0 26037713

[18] Tada T Kumada T Toyoda H Sone Y Kaneoka Y Maeda A Utility of combined gray-scale and perflubutane contrast-enhanced ultrasound for diagnosing early hepatocellular carcinomas: comparison of well differentiated and distinctly nodular types. Hepatol Res. (2016) 46(12):1214–25. 10.1111/hepr.12670 26860925

[19] Tada T Kumada T Toyoda H Ito T Sone Y Kaneoka Y Utility of contrast-enhanced ultrasound with perflubutane for diagnosing the macroscopic type of small nodular hepatocellular carcinomas. Eur Radiol. (2014) 24:2157–66. 10.1007/s00330-014-3254-2 24952601

[20] Shin SK Choi DJ Kim JH Kim YS Kwon OS. Characteristics of contrast-enhanced ultrasound in distinguishing small (≤3cm) hepatocellular carcinoma from intrahepatic cholangiocarcinoma. Medicine (Baltimore). (2018) 97(41):e12781. 10.1097/MD.0000000000012781 30313099 PMC6203535

[21] Shin SK Kim YS Choi SJ Shim YS Jung DH Kwon OS Contrast-enhanced ultrasound for the differentiation of small atypical hepatocellular carcinomas from dysplastic nodules in cirrhosis. Dig Liver Dis. (2015) 47(9):775–82. 10.1016/j.dld.2015.05.001 26043653

[22] Giorgio A Calisti G Di Sarno A Farella N De Stefano G Scognamiglio U Characterization of dysplastic nodules, early hepatocellular carcinoma and progressed hepatocellular carcinoma in cirrhosis with contrast-enhanced ultrasound. Anticancer Res. (2011) 31(11):3977–82. 22110230

[23] Zhang L Gu J Li Y Ren Z Zhang B Yu Z. Clinical value study on contrast-enhanced ultrasound combined with enhanced CT in early diagnosis of primary hepatic carcinoma. Contrast Media Mol Imaging. (2022) 2022. 10.1155/2022/7130533 PMC9462989 36101800

[24] Ye T Shao SH Ji K Yao SL. Evaluation of short-term effects of drug-loaded microspheres and traditional transcatheter arterial chemoembolization in the treatment of advanced liver cancer. World J Gastrointest Oncol. (2022) 14(12):2367. 10.4251/wjgo.v14.i12.2367 36568947 PMC9782616

[25] Riviere DM Van Geenen EJ Van Der Kolk BM Nagtegaal ID Radema SA Van Laarhoven CJ Improving preoperative detection of synchronous liver metastases in pancreatic cancer with combined contrast-enhanced and diffusion-weighted MRI. Abdom Radiol. (2019) 44:1756–65. 10.1007/s00261-018-1867-7 30659309

[26] Garcia-Carbonero R JImenez-Fonseca P Teulé A Barriuso J Sevilla I. SEOM clinical guidelines for the diagnosis and treatment of gastroenteropancreatic neuroendocrine neoplasms (GEP-NENs) 2014. Clin Transl Oncol. (2014) 16:1025–34. 10.1007/s12094-014-1214-6 25183048 PMC4239790

[27] Huang J Chen W Yao S. Assessing diagnostic value of contrast-enhanced ultrasound and contrast-enhanced computed tomography in detecting small hepatocellular carcinoma: a meta-analysis. Medicine (Baltimore). (2017) 96(30):e7555. 10.1097/MD.0000000000007555 28746202 PMC5627828

[28] Lan D Qu HC Li N Zhu XW Liu YL Liu CL. The value of contrast-enhanced ultrasonography and contrast-enhanced CT in the diagnosis of malignant renal cystic lesions: a meta-analysis. PLoS One. (2016) 11(5):e0155857. 10.1371/journal.pone.0155857 27203086 PMC4874594

[29] Schellhaas B Strobel D. Tips and tricks in ContrastEnhanced ultrasound (CEUS) for the characterization and detection of liver malignancies. Ultraschall Med. (2019) 40:404–24. 10.1055/a-0900-3962 31382313

[30] Li X Han X Li L Su C Sun J Zhan C Dynamic contrast-enhanced ultrasonography with sonazoid for diagnosis of microvascular invasion in hepatocellular carcinoma. Ultrasound Med Biol. (2022) 48(3):575–81. 10.1016/j.ultrasmedbio.2021.11.005 34933756

[31] Watanabe Y Ogawa M Kumagawa M Hirayama M Miura T Matsumoto N Utility of contrast-enhanced ultrasound for early therapeutic evaluation of hepatocellular carcinoma after transcatheter arterial chemoembolization. J Ultrasound Med. (2020) 39(3):431–40. 10.1002/jum.15118 31436341

[32] European Association for the Study of the Liver. EASL clinical practice guidelines: management of hepatocellular carcinoma. J Hepatol. (2018) 69:182–236. 10.1016/j.jhep.2018.03.019 29628281

[33] Jang HJ Kim TK Burns PN Wilson SR. Enhancement patterns of hepatocellular carcinoma at contrast-enhanced US: comparison with histologic differentiation. Radiology. (2007) 244:898–906. 10.1148/radiol.2443061520 17709836

[34] Kong WT Ji ZB Wang WP Cai H Huang BJ Ding H. Evaluation of liver metastases using contrast-enhanced ultrasound: enhancement patterns and influencing factors. Gut Liver. (2016) 10:283–7. 10.5009/gnl14324 26586554 PMC4780459

[35] Page MJ McKenzie JE Bossuyt PM Boutron I Hoffmann TC Mulrow CD The PRISMA 2020 statement: an updated guideline for reporting systematic reviews. Int J Surg. (2021) 88:105906. 10.1016/j.ijsu.2021.105906 33789826

[36] Ouzzani M Hammady H Fedorowicz Z Elmagarmid A. Rayyan—a web and mobile app for systematic reviews. Syst Rev. (2016) 5. 10.1186/s13643-016-0384-4 27919275 PMC5139140

[37] Zhang Z Ma C Luo Y. Diagnostic value of liver contrast-enhanced ultrasound in early hepatocellular carcinoma: a systematic review and meta-analysis. J Gastrointest Oncol. (2023) 14(2):626. 10.21037/jgo-23-211 37201077 PMC10186536

